# Electroacupuncture for the prevention of postoperative gastrointestinal dysfunction in participants undergoing vascular laparotomy under general anesthesia: a randomized controlled trial

**DOI:** 10.1186/s13020-016-0122-9

**Published:** 2017-01-16

**Authors:** Meng-yue Liu, Cheng-wei Wang, Zhou-peng Wu, Ning Li

**Affiliations:** 1 Department of Integrated Traditional Chinese and Western Medicine, West China Hospital of Si-chuan University, Chengdu, Sichuan China; 2 Department of Liver and Vascular Surgery, West China Hospital of Si-chuan University, Chengdu, Sichuan China

## Abstract

**Background:**

Postoperative gastrointestinal dysfunction (PGD) is a common complication following laparotomy under general anesthesia (GA). Abdominal distension occurs in 8–28% of surgeries within 24 h postoperatively. The present study aimed to analyze the efficacy of electroacupuncture (EA) for the prevention of PGD by applying preoperative EA stimulation of PC6 (*Neiguan*), ST36 (*Zusanli*), and ST37 (*Shangjuxv*) bilaterally twice within 24 h prior to surgery, compared with no acupuncture treatment.

**Methods:**

The study participants were assessed and selected from participants undergoing vascular laparotomy under GA at the Liver and Vascular Surgery Unit in West China Hospital of Sichuan University. The selected participants were randomly allocated to two groups: routine-treatment (RT) and EA group receiving EA at PC6, ST36, and ST37. A computer-generated list of random numbers was used to determine the allocation of the participants, with numbered opaque sealed envelopes containing the randomization schedule. Eligible participants were all adults aged 18 years or above who were scheduled to undergo vascular laparotomy under GA within 24 h and had no history of EA treatment. The exclusion criteria included participants with serious systemic disease and history of EA treatment. While the RT group received standard treatments, the EA group received additional EA treatments. During each treatment session, EA stimulation was performed for a duration of 20 min at a frequency of 15 Hz with a continuous wave. All such participants received two EA treatments within 24 h before surgery. The outcomes were measured in three metrics: incidence and degree of abdominal distension; first times of flatus and defecation; and duration of hospitalization.

**Results:**

Forty-three participants were recruited, of whom 42 participants successfully completed the study. Each group contained 21 participants. The incidence of abdominal distension (42.8, 76.2%) and degree of abdominal distension were significantly reduced in the EA group (*P* = 0.03 and *P* = 0.03, respectively). In comparisons of the first times of flatus (3.05 ± 0.58, 3.29 ± 0.42 days) and defecation (2.81 ± 0.51, 3.20 ± 0.55 days) and duration of hospitalization (5.33 ± 0.68, 5.75 ± 0.66 days), the EA group was superior to the RT group to some extent (*P* = 0.13, *P* = 0.02, and *P* = 0.04, respectively).

**Conclusions:**

Preoperative EA at PC6, ST36, and ST37 might be useful for preventing PGD, thereby improving gastrointestinal function recovery.

*Trial registration* This study is registered with the Chinese Clinical Trial Registry: ChiCTR-TRC-13003649

**Electronic supplementary material:**

The online version of this article (doi:10.1186/s13020-016-0122-9) contains supplementary material, which is available to authorized users.

## Background

Postoperative gastrointestinal dysfunction (PGD) is a complication in participants undergoing major surgical procedures under general anesthesia (GA) [1]. Opioid-induced gastrointestinal dysfunction is as high as 81% in the United States [2]. Abdominal distension within 24 h of surgery occurs in 8–28% of all operations [3]. PGD causes longer hospital stays, greater medical cost [4], and higher risks for other complications, such as infections. In China, the rate of PGD remained the same between 1974 and 2011 [5]. In Japan, the rate increased by 10% over a 5-year period at the turn of the twenty-first century [6]. Internationally, an analysis was conducted on the records of more than 5000 participants undergoing major vascular surgery in 206 National Surgical Quality Improvement Program hospitals from 2008 to 2009 [7]. The results showed that >25% of participants with endovascular treatment, regardless of severity, suffered from postoperative complications, postoperative intestinal obstruction, and other gastrointestinal dysfunctions within 30 days of surgery. Although the 30-day survival rate of thoracoabdominal aortic operations was 92% [8], gastrointestinal dysfunction was ranked 4th among the causes for death [9].

Currently, there are four major methods of treatment for PGD: diet and nutritional support, drug treatment, operative procedure, and complementary medicine. Most of these treatments are open-label treatment trials with small samples [10]. Acupuncture is widely used in obstetrics and gynecology [11], gastrointestinal surgery [12], and orthopedics [13].

As a potential alternative therapy, acupuncture has gradually gained acceptance from physicians following the demonstration of its efficacy in clinical and basic research. For example, a 165-patient clinical study demonstrated that electroacupuncture (EA) reduced the duration of operative ileus [14]. Another small-sample-size trial found EA useful for postoperative recovery of abdominal distension [15], and a similar study showed that acupressure improved the recovery of stomach distension [16]. Animal experiments identified the factors contributing to PGD, including reduction in the number of interstitial cells of Cajal and atrophy of their structure [17], activation of the sympathetic nervous system and postoperative inflammatory cytokines [18], and leukocyte cell-induced nitrous oxide release, which restrained gastrointestinal peristalsis and causes intestinal inflammation [19]. Other animal studies showed that acupuncture ameliorated PGD by regulating these pathological changes [20].

The present study aimed to analyze the efficacy of EA for preventing PGD by applying preoperative EA stimulation of PC6 (*Neiguan*), ST36 (*Zusanli*), and ST37 (*Shangjuxv*) bilaterally twice within 24 h prior to surgery, compared with no acupuncture treatment.

## Methods

### Ethics approval

This study was approved by the Ethics and Research Committee of West China Hospital of Sichuan University (WCHSU), China [No. 2013 (105); Additional file 1]. The Ethics and Research Committee of WCHSU is an ethical approval committee organized by leading professors in their own fields. The study strictly followed the principles for medical ethics outlined in the Declaration of Helsinki [21]. The study was registered with the Chinese Clinical Trial Registry: ChiCTR-TRC-13003649. All participants were provided with information regarding the study and signed a written consent form in advance (Additional file 2). The Chinese Cochrane Center was responsible for the whole trial including the randomization, blinding, statistical analyses, and data management. The study was performed according to the CONSORT 2010 checklist (Additional file 3).

### Trial design

This was a single-center, single-blind, pragmatic randomized controlled study. With balanced randomization (1: 1), the participants were assigned to two groups: EA group and RT group (routine-treatment group).

### Participants

All participants were recruited from the WCHSU, China, after meeting the eligibility criteria and signing the informed consent forms. Chengdu is a major business city in the west of China, with a population of 14,047,600, and WCHSU is the primary health care provider in the west of China. Eligible participants were all adults aged 18 years or above who were scheduled to undergo vascular laparotomy under GA within 24 h and had no history of EA treatment.

The exclusion criteria were as follows: participants undergoing endovascular surgery; participants with a history of hypothyroidism, hyperthyroidism, cardiopulmonary disease, diabetes, or psychological disorder; participants with a history of EA treatment; participants with a cardiac pacemaker; participants in the menstruating phase of the menstrual cycle; participants who refused to accept acupuncture; participants who were unconscious before surgery; participants with inability to communicate; participants participating in another clinical trial that could interfere with the primary endpoint of the study; participants with a bleeding disorder (hemophilia or fibrinogenemia); participants with serious systemic disease (AIDS or sepsis); participants with initial body temperature of above 38.0 °C or below 36.0 °C; participants with a known history of alcohol or substance abuse; participants requiring systemic sedation for other reasons; participants needing emergency procedures; participants who were pregnant or lactating women.

The withdrawal criteria were as follows: at the patient’s own request or at the request of their legal representative; if, in the opinion of the investigator or the specialized physician performing the acupuncture or examination, continuation of the trial would be detrimental to the patient’s wellbeing (e.g. strong pain at insertion points, allergic reactions, other independent acute health problems).

### Interventions

For prevention of PGD by EA, three acupuncture points, PC6 (*Neiguan*), ST36 (*Zusanli*), and ST37 (*Shangjuxv*), were selected.

**Fig. 1:**
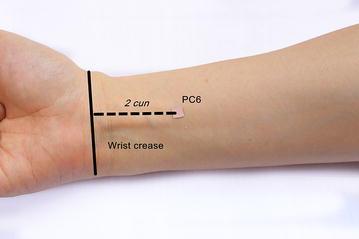
Location of PC6

**Fig. 2:**
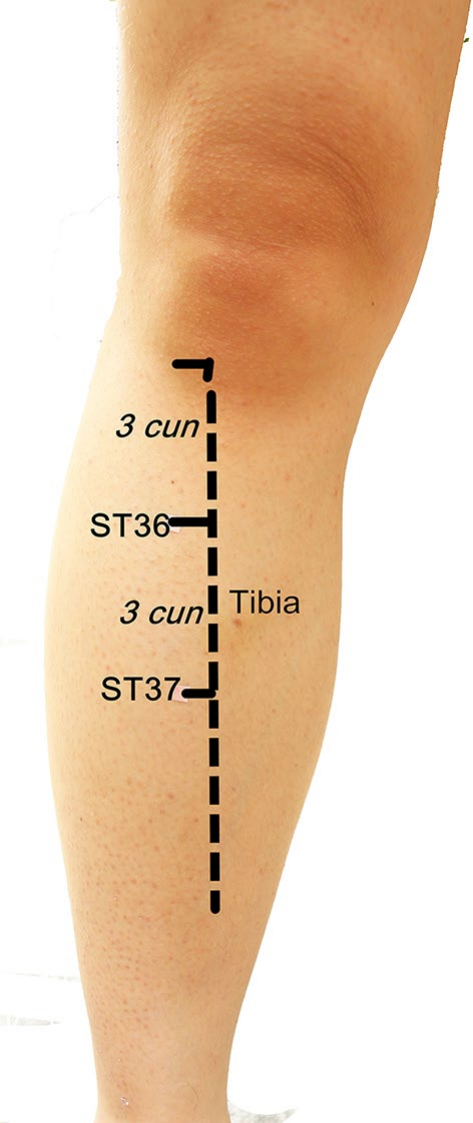
Locations of ST36 and ST37

The participants receiving EA therapy were treated bilaterally at the above three distal acupuncture points. PC6 is an acupuncture point of the pericardium meridian, which is located between the palmaris longus tendon and the flexor carpi radialis muscle tendon, 2 cun above the rasceta (Fig. 1). ST36 in a point between the tibialis anterior and extensor digitorum longus, which is located on the anterolateral side of the lower leg, 3 cun below *Dubi* and one finger width lateral of the anterior border of the tibia. ST37 is a point in the tibialis anterior, which is located 3 cun below ST36 (Fig. 2). The anterior tibial artery and anterior tibial vein are present around both ST36 and ST37, while there are the lateral sural cutaneous nerve and cutaneous branch of the saphenous nerve. The locations of these acupuncture points are described in *The National Standards for Acupoint Location* [22].

The participants in EA group were given the following EA treatment. After each insertion site was disinfected with 75% alcohol, sterile single-use 0.25 × 38-mm needles (Wuxi Jiajian Medical Instrument Co. Ltd., Jiangsu, China) were inserted vertically to a depth of 4–10 mm. At the appropriate depth, the acupuncturist manipulated the needles for *Deqi* sensation, defined as a feeling of heaviness around the acupoint [23], and then bilaterally connected a Nerve and Muscle Stimulator (Suzhou Medical Appliance Factory, Suzhou, China) to PC6 (positive electrode) and ST36 (negative electrode) on the ipsilateral limbs. In each treatment session, EA stimulation was performed for a duration of 20 min at a frequency of 15 Hz with a continuous wave. The participants received two EA treatments within 24 h before surgery, one at 10:00 a.m. and another at 16:00 p.m.

All participants in 2 groups received similar routine treatments after surgery, including gastrointestinal decompression, maintenance stabilization of homeostasis (especially maintenance of electrolyte balance), routine nursing care, and early walking, with no further EA treatments in EA group. The only combination therapy received by all participants was 1500 mL of physiological saline with 1.5 g Potassium Chloride Injection (China Otsuka Pharmaceutical Co. Ltd., Tianjin, China) for 2 days postoperatively.

**Fig. 3:**
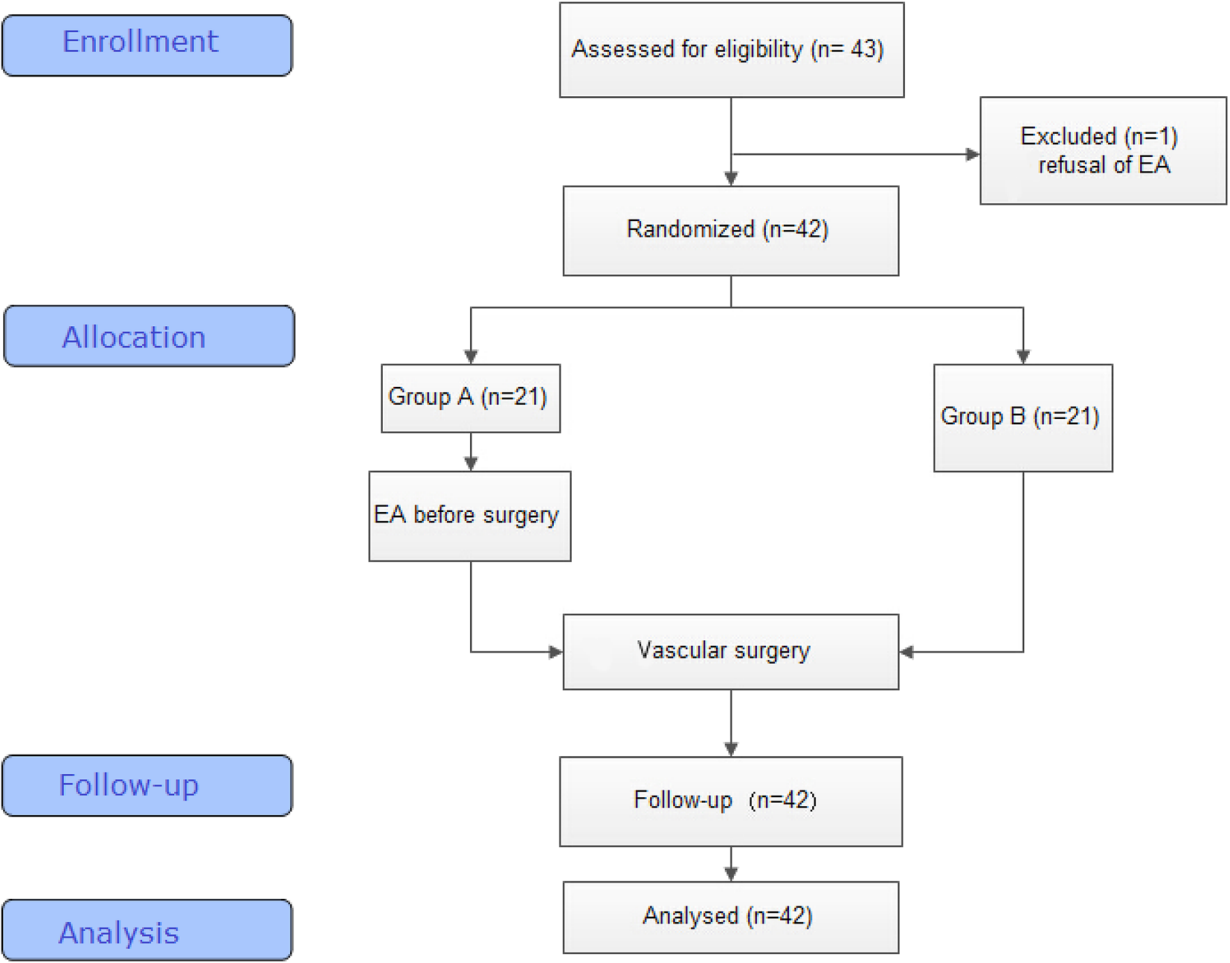
Flowchart of the study

All participants underwent preoperative and postoperative supervision and evaluation of curative effects by a blinded observer at 10:00 a.m. daily, until they were discharged. A third party performed all data treatments and analyses. The study was performed as shown in the flowchart (Fig. 3).

The participating acupuncturist had more than 2000 h of acupuncture training before participating in the trial and received their diploma 6 years ago. The physician had applied acupuncture in practice for more than 6 years and had treated about 1000 participants with acupuncture in the year before the trial.

### Outcomes

A blinded observer obtained the postoperative data including the degree of abdominal distension, times of first flatus and defecation, and duration of hospitalization after surgery. As a subjective sensation, abdominal distension was measured by a Likert-type scale (Table 1) [24]. Postoperative abdominal distension was defined as a postoperative gastrointestinal motility change leading to invalid transfer of gastrointestinal contents [25], caused by intraoperative wound traction, abdominal adhesions, or lack of visceral perfusion [2]. In this study, the incidence and degree of abdominal distension after surgery were the main outcome indicators, while the times of first flatus and defecation and duration of hospitalization were secondary outcomes. The study focused on the prevention of PGD; therefore, the incidence of PGD was the primary endpoint.

**Table 1:** Presentation of the Likert-type scale

Score	Explanation
0	I feel no abdominal distension at all
1	I feel a little of abdominal distension
2	I feel abdominal distension but I can bear
3	I feel obvious abdominal distension that I cannot bear but it doesn’t affect my life
4	I feel horrible abdominal distension and need it addressed

The study included a period from 24 h preoperatively to 7 days postoperatively. A graduate student, who did not participate in patient management, recorded the treatment details, including demographic data, anesthesia time, surgery duration, and dosage of opioids. The demographic data collected included: age; sex; race; marital status; job status; educational background; weight; and telephone number. Considering that the type of intravenous fluid given during and after surgery is a factor in postoperative recovery, we specified that all participants enrolled in the study were only under GA perioperatively and that all of their postoperative routine treatments, especially the combination therapy, were the same to avoid any bias conferred by administration of a different intravenous fluid.

### Sample size calculation

Owing to a lack of previous reports on PGD-related acupuncture and vascular surgery, the study was designed to include a minimum of 20 cases [26] in each group following the minimum sample-size requirements. With consideration of selecting an appropriate sample size, controlling random errors, and achieving a certain effect, the lower limit of the sample size was set at 20 cases (20–30 cases per group stipulated by the China Food and Drug Administration, 20–50 cases in the European Union, 20–80 cases in the United States [26]).

### Randomization and blinding

All participants were randomly assigned to the two groups on the basis of a concealed allocation approach using statistical analysis PROCPLAN statements (SAS 9.4; World Headquarters SAS Institute Inc., Cary, NC, USA). A computer-generated list of random numbers was used to determine the allocation of the participants, with numbered opaque sealed envelopes containing the randomization schedule. The envelopes were kept by an investigator who was not an assessor in the study, and who was informed of the outcomes at the end of the study. The researchers recording the outcomes and those making the conclusions were all blinded to patients’ assessments.

### Statistical analysis

All continuous data, including the incidence of abdominal distension, all secondary outcomes, and some of the baseline data, were expressed as mean ± standard deviation (SD). For these data, a *t* test used to compare normally distributed data between the two groups, while the Wilcoxon rank sum test applied for non-normally distributed data. Nominal variables, including the degree of abdominal distension and the remaining baseline data, were presented as frequency and percentage and analyzed using the Chi square (χ^2^) test. Values of *P* <0.05 were considered statistically significant. All the efficacy and safety analyses were performed using the intent-to-treat population. A third person performed blinded estimation and statistics of the curative effect.

### Adverse events

All adverse events, such as hematoma, stuck needle, and fainting, were reported. In participants with serious adverse events, the experimental intervention was ceased immediately, and proper treatments were provided.

## Results

### Selection of participants

Recruitment for the study began on 10 February 2014 and finished on 14 July 2014, while follow-up was completed on 17 July 2014. During the 8-month-study, 43 participants were recruited, and 42 participants completed the study, with 21 participants in each group. One participant was excluded because of refusal to receive EA. The baseline demographic data and clinical characteristics of the two groups did not differ significantly and were comparable (Table 2). The mean morbidity age was 56.40 ± 11.61 years, and the participants comprised 23.8% women and 76.2% men.

**Table 2:** Demographic data for all participants

	EA group (N = 21)	RT group (N = 21)	*t*	χ^2^	*P*
Age (years), mean ± SD	56.0 ± 11.31	56.8 ± 12.16	0.221		0.826
Gender (F), %	23.8%	23.8%	–	0.130	0.641
Race (Han), %	90.5%	85.7%	–	0.000	0.500
Married, %	95.2%	100%	–	0.000	0.235
Retirement, %	23.8%	23.8%	–	0.000	0.280
Illiteracy/yes, %	4.8%	19.0%	–	0.980	0.472
Weight (kg), mean ± SD	65.6 ± 2.56	64.7 ± 1.16	1.467	–	0.150
Anesthesia time (h), mean ± SD	4.03 ± 0.35	4.19 ± 0.38	1.419	–	0.164
Surgery duration (h), mean ± SD	3.52 ± 0.28	3.43 ± 0.31	0.987	–	0.329
Opioids’ dosage (g), mean ± SD	0.86 ± 0.48	0.67 ± 0.66	1.067	–	0.292

*EA* electroacupuncture, *RT* routine treatment

### Outcomes and estimations

#### Primary endpoints

As shown in Table 3, there were significant differences in the incidence and degree of abdominal distension between the two groups (*P* = 0.03 and *P* = 0.03, respectively). The percentage of participants suffering from abdominal distension was 42.8% in EA group and 76.2% in RT group. The number of participants who could not stand the distension was 14.3% in EA group and 33.3% in RT group, as well as 28.6% in EA group and 37.9% in RT group were with sustainable abdominal distension which don’t need any pharmacotherapy.

**Table 3:** Outcomes for incidence and degree of abdominal distension

	EA group (N = 21)	RT group (N = 21)	*t*	Z	*P*
AD’s incidence	9 (42.8%)	16 (76.2%)	4.842		0.029
AD’s degree				−2.109	0.035
0	12 (57.1%)	5 (23.8%)			
1	0 (0%)	2 (4.6%)			
2	6 (28.6%)	7 (33.3%)			
3	3 (14.3%)	4 (19.0%)			
4	0 (0%)	3 (14.3%)			

Data are shown as *n* (%)

*EA* electroacupuncture, *RT* routine treatment, *AD* abdominal distension

#### Secondary endpoints

As shown in Table 4, the time of first defecation (2.81 ± 0.51, 3.20 ± 0.55 days) and duration of hospitalization (5.33 ± 0.68, 5.75 ± 0.66 days) showed significant differences between the two groups (*P* = 0.02 and *P* = 0.04, respectively), while the time of first flatus (3.05 ± 0.58, 3.29 ± 0.42 days) showed no significant difference (*P* = 0.13). Among the three items, the reduction in first defecation time was most noticeable (12.18%), while the time of first flatus, showing the beginning of gastrointestinal function recovery, was reduced by 7.29% and the duration of hospitalization was shortened by 6.98%.

**Table 4:** Outcomes of the secondary endpoints

	EA group (N = 21)	RT group (N = 21)	*t*	*P*
First time flatus (d)	3.05 ± 0.58	3.29 ± 0.42	1.536	0.132
First time Defecation (d)	2.81 ± 0.51	3.20 ± 0.55	2.383	0.022
Hospitalization (d)	5.33 ± 0.68	5.75 ± 0.66	2.031	0.049

Data are shown as mean ± SD

*EA* electroacupuncture, *RT* routine treatment, *AD* abdominal distension

#### Adverse events

There were no participants with needle breakages, unbearable pain, and other acupuncture-related adverse events during the study.

## Discussion

This study was carried out to determine whether a preventive intervention program can reduce PGD after vascular surgery under GA. In WCHSU, gastrointestinal decompression or early walking is usually used for treatment of PGD, and these methods were applied to all study participants. For prevention of PGD, a previous study stated that EA should be applied from anesthesia induction to surgery completion [27]. However, application of EA was chosen during the 24-h period before surgery for four reasons: research purposes; safety; easy isolation of control and treatment groups; and efficacy. As a pre-validation study, the present study focused on whether this preventive intervention had an impact on PGD, while rehabilitation or acupuncture is often prescribed after surgery. In addition, this study used EA, which may interfere with other equipment used intraoperatively, such as an electric knife. In traditional Chinese medicine theory and human research [28], *Deqi* is a prerequisite for effective treatment. During or after surgery, GA and postoperative incision pain can cause a needling sensation, resulting in reduced levels of patient compliance.

The anesthetic items for both groups were compared, and only laparotomy was included to mitigate the experimental bias caused by different operative methods and anesthetic concentrations, especially opioids [29]. As the anesthetic concentration was proportional to weight, and there was no significant difference in weight between the two groups, the anesthetic dosages for both groups were comparable.

All secondary outcomes, used extensively for evaluating functional recovery, indicated that EA could accelerate the rehabilitation program for PGD. Among the three endpoints, the reduction of first defecation time showed the largest effect. Studies in the literature have used varying endpoints, such as bowel sounds, flatus, and bowel movements [30, 31]. Among these three endpoints, bowel movements are the most reliable endpoint [32].

To mitigate the potential bias among consenting participants induced by the Hawthorne effect, many studies have required inclusion of a sham acupuncture group. There are four methods in common use [33], as shown in Table 5. However, researchers rarely use placebo-controlled trials when comparing interventions with general routine therapy [34]. Acupuncture treatments are complex, multicomponent interventions. In sham-controlled trials that attempt to control only certain potentially therapeutic acupuncture-specific components, such as location, insertion depth, stimulation, needle size, and number [35], the moderately large nonspecific effects of sham acupuncture can decrease the reliability and the usefulness of the test results [36].

**Table 5:** Descriptions of the four common methods of sham-acupuncture

Advantages	Limitations	Indications
Sham-acupuncture		
Fine masking	Not suitable	Insistent estimation
Low stimulation	For all acupoints	Without EA history
		Acupoints on back
Non-acupoints		
Fine masking	Can’t avoid	Different ganglion segment
Better feasibility	Physiological effect	Without EA history
		Study on acupoints’ specificity
Shallow acupuncture		
Better masking	Narrow indication	Study on depths of needling
Low stimulation		
Non-disease-related acupuncture		
Better masking	Low feasibility	Without EA history
Lowest bias		Study on acupoints’ specificity

The incidence and degree of abdominal distension determined that EA was useful for preventing PGD and helped in the drawing of accurate conclusions. The Likert-type scale was used to measure the degree of abdominal distension, and the sustainable uncomfortable, which don’t influent sleep, is 1′–2′ and the uncomfortable need pharmacotherapy is 3′–4′. In our study, EA could alleviate abdominal distension more. However, the Likert-type scale is only a rough estimation, although there is no specialized abdominal-distension personnel. Therefore, regarding validity of the findings, a comparison of objective parameters is necessary in a future study. The latest animal and human studies have demonstrated that not only a leukocytic pathway and subsequent injury-specific local inflammatory responses [37, 38], but also secondary lymphoid organs 0, are involved in the progression of postoperative ileus. It is indispensable to add an inflammatory cytokine test to a future study. There is also a necessity for a comparison of abdominal circumference as an objective factor 0.

There is a bias to the present study that cannot be ignored, namely that EA group had more doctor–patient contact hours and might experience placebo effects, even though the acupuncturist was not their surgeon. Considering that the acupuncturist performed EA only twice, the acupuncturist was recommended to get in contact with the participants in RT group in a future study.

## Conclusions

Preoperative EA at PC6, ST36, and ST37 might be useful for preventing PGD, thereby improving gastrointestinal function recovery.

## Additional files

**Additional file 1.** The ethic approval.

**Additional file 2.** The informed consent.

**Additional file 3.** The checklist.

## Abbreviations

EA: electroacupuncture
GA: general anesthesia
PGD: postoperative gastrointestinal dysfunction
WCHSU: West China Hospital of Sichuan University
