# Supplementary material for: Electroacupuncture for the prevention of postoperative gastrointestinal dysfunction in participants undergoing vascular laparotomy under general anesthesia: a randomized controlled trial
Source: Chin Med. 2017 Jan 16;12:5. doi: 10.1186/s13020-016-0122-9 (PMC5240264; doi:10.1186/s13020-016-0122-9)
Supplement: Supplementary file 1 — Additional file 1. The ethic approval. [file 13020_2016_122_MOESM1_ESM.pdf]

四川大学华西医院临床试验与生物医学伦理专委会审查批件

2013年 审（105）号

科室（专业）：中西医结合科		项目负责人姓名及职称：李宁　副教授	
项目名称	电针对防治血管外科行全麻术后患者胃肠功能紊乱的临床观察研究		
研究方案	版本号：无		版本日期：2013.9.17
知情同意书	版本号：第1版		版本日期：2013.7.16

审查意见：

1. 研究者资质符合伦理要求。
2. 研究方案及知情同意书基本符合伦理要求。

审查结果：■同意　□作必要修正后同意　□作必要修正后再审　□不同意　□终止或暂停

请遵循我国相关法律、法规和规章（SFDA《药物临床试验质量管理规范》（2003）、《医疗器械临床试验规定》（2004）、WMA《赫尔辛基宣言》和CIOMS《人体生物医学研究国际道德指南》、卫生部《涉及人的生物医学研究伦理审查办法（试行）（2007）》），遵循伦理委员会批准的方案和知情同意书开展临床试验（研究），保护受试者的健康与权利。

在试验（研究）过程中，若变更主要研究者，对临床研究方案、知情同意书等的任何修改，请申请人提交修正案审查申请。

发生严重不良事件，请申请人及时提交严重不良事件报告；紧急报告之后，尽快提交详细的严重不良事件随访报告。

请递交年度和定期跟踪审查报告；当出现任何可能显著影响试验（研究）进行或增加受试者危险的情况时，请申请人及时向伦理专委会提交书面报告。

试验（研究）纳入了不符合纳入标准或符合排除标准的受试者，符合中止试验（研究）规定而未让受试者退出试验（研究），给予错误治疗或剂量，给予方案禁止的合并用药等没有遵从方案开展研究的情况；或可能对受试者的权益/健康、以及研究的科学性造成不良影响等违背伦理原则与规范的情况，请申办者/监查员/研究者提交违背方案报告。

申请人暂停或提前终止临床试验（研究），请及时提交暂停/终止试验（研究）报告。完成临床试验（研究），请申请人提交结题报告。

单 位（章）：

主任委员（签名）：[illegible]

2013 年 9 月 26 日
