# Supplementary material for: Electroacupuncture for the prevention of postoperative gastrointestinal dysfunction in participants undergoing vascular laparotomy under general anesthesia: a randomized controlled trial
Source: Chin Med. 2017 Jan 16;12:5. doi: 10.1186/s13020-016-0122-9 (PMC5240264; doi:10.1186/s13020-016-0122-9)
Supplement: Supplementary file 2 — Additional file 2. The informed consent. [file 13020_2016_122_MOESM2_ESM.docx]

**电针防治血管外科全麻术后患者胃肠功能紊乱的临床观察研究知情同意书**

**亲爱的患者：**

我们将邀请您参加一项电针防治血管外科全麻术后患者胃肠功能紊乱的临床观察研究。本研究的目的是：观察电针早期介入对预防全麻术后出现胃肠功能紊乱的临床治疗效果，为临床提供更多的循证证据。

**1、研究介绍**

血管外科全麻下行血管腔内治疗并发胃肠功能紊乱，又称术后胃肠道并发症，是手术后的最常见并发症之一，超过半数以上的腹部手术患者均会导致该并发症出现，可导致住院日严重延长等。

基础研究发现，肠道内Cajal间质细胞的数量减少和结构萎缩、信号转导功能下降是该并发症发生的基础病理变化，而术后交感神经兴奋，炎症因子释放以及胃肠激素分泌紊乱也可导致该并发症的发生。有动物模型试验证实针刺可使Cajal细胞结构趋于正常，同时实验针灸学证实针刺可通过增加血管紧张素及一氧化氮的水平来增强胃肠蠕动,改善胃肠血流分布,调节胃动素和生长抑素含量的功能，具有预防及治疗术后胃肠功能紊乱作用。

**2、临床观察方法**

本研究采用前瞻性随机对照试验方法，预计有140名患者自愿参加。若您符合纳入标准并愿意参加本研究，将按以下步骤进行研究：

**（1）血管外科专科医师采取对症、支持与防治并发症的积极术后介入治疗。**

（2）针灸医生将根据计算机提供的随机数字，决定您分在电针组或对照组。

（3）本研究中使用的针灸针为无锡佳健医疗器械有限公司生产的佳健牌一次性针灸针，生产企业许可证：苏食药监械生产许2006-0095号，注册证号：苏食药监械（准）字2011第2270162号。规格是φ0.25×40mm，即粗细规格为0.25mm，长短规格为1.5寸。使用的电针仪为江苏医用设备厂生产的华佗牌SDZ-V电针仪。

（4）研究采用的电针频次和疗程：12小时1次；每次20分钟。

（5）研究期间医生将收集您的病史及体检结果，包括：腹胀是否发生、发生的程度、腹围大小、首次排气时间、首次排便时间、术后住院天数等，每次检查的内容和具体步骤，您的医生将负责给予说明和指导。

**3、受试者的权利**

如果您参加本研究，在研究期间，将得到免费针灸治疗及术后健康知识支持。

到目前为止，尽管没有发现针灸，尤其是电针治疗有轻度以上不良反应。但针刺过程中您可能会有酸、麻、重、胀等感觉，这均为针刺的正常反应。针刺治疗可能存在不良反应，但较少而轻微，针刺时可能因为您的体质问题或情绪紧张出现晕针现象，停止针灸和适当休息后可缓解；针刺后可能出现出血、血肿等现象，经局部按压后可消失；您有权决定参加或拒绝参加本试验，且任何时侯都可以退出。无论何种原因，您退出本试验，都不会影响您和医生间的医患关系，您仍将得到及时的治疗。

出于对您的最大利益考虑，医生或研究者可能会在研究过程中随时中止您继续参加本项研究。如果在研究过程中有任何重要的新信息，可能影响您继续参加研究的意愿时，您的医生将会及时通知您。

您有权在任何时间咨询问题。是否参加研究完全取决于您的意愿。您可以拒绝参加此项研究，或在研究过程中的任何时间退出本研究，这都不会影响您和医生间的关系，都不会影响对您的医疗或有其他方面利益的损失。

伦理委员会已经审查此项研究符合科研医德，并在赫尔辛基宣言指导下进行。

签署了这份知情同意书，即表示您同意参加本试验。您的试验资料将记录在病例报告表上。您的姓名不会出现在任何研究报告和公开出版物中。您与本试验相关资料都将得到严格保密及进行可信的统计处理。您的医疗记录将由权威机构评定。在必要情况下，只有申办者、研究者、监查员、伦理委员会有权查阅您所有的试验记录资料，而其他人均无权接触。

━━━━━━━━━━━━━━━━━━━━━━━━━━━━━━━━━━━━━━━

受试者承诺：

**我已阅读了知情同意书告知的内容，并且完全理解。我的医生已给我做了有关本研究的完整的解释，我确认已有充足的时间考虑，所有的疑问都已得到圆满的解答。我同意自愿参加本试验，并愿意接受按要求与研究者合作完成本试验。**

受试者签名： 电话：

日期： 年 月 日

研究者承诺：

**我确认已向受试者解释了本研究的详细情况，包括可能的获益和风险。**

研究者签名： 电话

日期： 年 月 日
